# Tailoring and personalizing deep brain stimulation for Parkinson's disease

**DOI:** 10.1055/s-0044-1786823

**Published:** 2024-05-19

**Authors:** Rubens Gisbert Cury, Carina França

**Affiliations:** 1 Universidade de São Paulo, Faculdade de Medicina, Departamento de Neurologia, Centro de Distúrbios do Movimento, São Paulo SP, Brazil; 2 Hospital Israelita Albert Einstein, São Paulo SP, Brazil

While numerous studies over the past three decades have clarified that deep brain stimulation (DBS) is safe and effective for treating Parkinson's disease (PD) symptoms, 1 it is not unusual to come across neurologists who are not yet acquainted with this technique. 2 Knowledge regarding DBS is not widely available in many neurology training centers, particularly in developing countries. This gap is in great part responsible for the disproportion between the number of PD patients to which surgery should be offered and the actual number of patients in which the surgery is actually and correctly performed. 2

In this issue of the journal, an article in two parts by Aquino and Moscovich et al. 3 4 explored the state-of-the-art and new and future technologies in neuromodulation for PD. The authors reviewed the DBS indication criteria, expected outcomes, and possible targets according to patients' profiles, programming, and medication management. They also explore areas of less consensus, such as DBS mechanisms of action and future technological improvements.

Although the benefits of DBS for PD have been well established, improvements in motor and non-motor symptoms and quality of life are intertwined with good patient selection. 5 The reason for this is that not all PD patients are good DBS candidates, and the ability to differentiate between good and bad candidates is paramount for DBS success. 6 As highlighted by Aquino et al., a good presurgical evaluation takes time and should be thorough. A patient should not be referred to DBS before being educated regarding possible benefits and risks and before a proper assessment, including the levodopa challenge test and psychiatric and cognitive aspects. The ability to maintain frequent medical appointments in a specialized center should also be taken into consideration, especially in countries with large geographical areas, such as Brazil. Moreover, it is important to note that DBS should only be offered to PD patients with more than 4 years of motor symptom onset and presents one of three indications: motor fluctuations, refractory tremor, or medication intolerance.

On top of this “classic” selection criteria, it is well known that genetic and imaging data are also important DBS outcome predictors, and, when available, should be taken into consideration. 7 8 Vascular changes, smaller middle and superior frontal cortical thickness, preoperative lower parieto-occipital glycolytic uptake, and higher primary motor cortex glycolytic uptake are some of the imaging findings that predict a worse motor outcome. 9 Although a minority of PD patients have genetic abnormalities, it is well known that PRKN mutation carriers have good motor outcomes and minimal cognitive decline, whereas GBA mutation carriers usually have worse cognitive and neuropsychiatric outcomes. Carriers of LRRK2 mutations may have different outcomes, depending on the specific type of mutation. 9 While these findings should not be the main reason to refer or exclude patients from a DBS referral, they should be considered as additional tools to help both patients and physicians manage surgical expectations.

Lead placement accuracy is one of the most important factors in establishing DBS success, after defining good and bad DBS candidates and deciding the best surgical target. In this regard, direct target visualization is greatly improved with ultra-high-field MRI (7T and above), although this technique is prone to higher distortion susceptibility. 9 Alternative MRI sequences can also help improve surgical planning, such as susceptibility weighted imaging (SWI), Fast Gray Matter Acquisition T1 Inversion Recovery (FGATIR), and Quantitative Susceptibility Mapping (QSM) applied to multi-echo gradient-recalled echo (GRE) acquisitions. 9 A novel approach to DBS targets is to think not about gray matter nuclei, but about networks. In this sense, tractography can provide additional information for surgical planning. 9

After good patient selection and adequate lead positioning, correct DBS programming is the final step responsible for good motor and non-motor outcomes. Considering that DBS programming is a time-consuming task, mainly based on electrophysiological and neuroanatomical notions, as well as trial-and-error, much effort is now being directed toward making this process quicker and easier. Recently, imaging tools have also helped neurologists perform this arduous task. Software such as Lead DBS and Brainlab Elements can accurately point to the lead location in each patient and estimate the volume of tissue activated with a set of chosen parameters, improving neuroanatomical visualization during DBS programming sessions and decreasing time spent, but with the same symptomatic control. 10 A recent study found non-inferiority of motor symptom control compared with programming based on StimFit, an algorithm capable of suggesting optimal stimulation parameters based on electrode location, and classic programming sessions. 11

DBS has completely changed the treatment of patients with PD and is currently considered responsible for a “second honeymoon” period, following the classic honeymoon phase (benefit from drug therapy in the early stages of the disease). While its effects on motor and non-motor symptoms (pain, sleep, sweating, and non-motor fluctuations) are life-changing, many challenges still remain. Some of these are linked to the poor knowledge of many healthcare professionals regarding patient selection and postoperative management. Others are due to therapy limitations, especially regarding cognition, speech, and gait, and recent efforts are being directed toward noninvasive modulation and new DBS targets. 12 13 Hopefully, innovations will generate further improvements in this blooming field.
